# Metformin inhibits inflammatory response and endoplasmic reticulum stress to improve hypothalamic aging in obese mice

**DOI:** 10.1016/j.isci.2023.108082

**Published:** 2023-09-27

**Authors:** Leilei Yang, Peng Lu, Xiangyu Qi, Qian Yang, Luna Liu, Tao Dou, Qingbo Guan, Chunxiao Yu

**Affiliations:** 1 Key Laboratory of Endocrine Glucose & Lipids Metabolism and Brain Aging, Ministry of Education; Department of Endocrinology, Shandong Provincial Hospital Affiliated to Shandong First Medical University, Jinan, Shandong, China; 2 Shandong Key Laboratory of Endocrinology and Lipid Metabolism, Jinan, Shandong, China; 3 Shandong Institute of Endocrine and Metabolic Diseases, Jinan, Shandong, China; 4 Shandong Engineering Laboratory of Prevention and Control for Endocrine and Metabolic Diseases, Jinan, Shandong, China

**Keywords:** Physiology, Molecular biology, Neuroscience, Immunology

## Abstract

The hypothalamus, as a vital brain region for endocrine and metabolism regulation, undergoes functional disruption during obesity.The anti-aging effect of metformin has come into focus. However, whether it has the potential to ameliorate hypothalamic aging and dysfunction in the obese state remains unclear. In this study, obese mice were utilized to investigate the effects of metformin on the hypothalamus of obese mice. According to the results, metformin treatment resulted in improved insulin sensitivity, reduced blood glucose and lipid levels, as well as attenuation of hypothalamic aging, demonstrated by decreased SA-β-gal staining and downregulation of senescence markers. Additionally, metformin decreased the expression of endoplasmic reticulum stress-related proteins in neurons and reduced the inflammatory response triggered by microglia activation. Further mechanistic analysis revealed that metformin inhibited the expression and activation of STING and NLRP3 in microglia. These results reveal a possible mechanism by which metformin ameliorates hypothalamic aging.

## Highlights

• Metformin ameliorates hypothalamic aging in obese mice
• Metformin inhibits hypothalamic microglia inflammation and neuronal ER stress
• The effect of metformin on hypothalamic aging is related to STING pathway in microglia

## Graphical abstract

**Figure:**
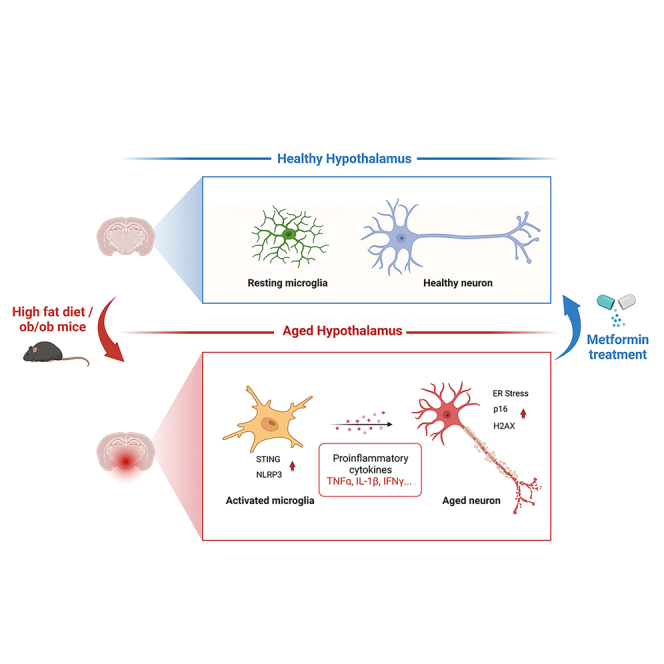

## Introduction

Obesity has become a global health concern. Previous studies have demonstrated that obesity contributes to accelerated aging in various tissues and organs,^1^ increasing the risk of well-established metabolic disorders, including type 2 diabetes and cardiovascular diseases. Additionally, studies based on magnetic resonance imaging and neurocognitive tests have revealed that obesity accelerates aging in the hippocampus, promoting cognitive impairments in young to middle-aged individuals.^2^ The hypothalamus, a vital brain region within the central nervous system (CNS), regulates various vital physiological processes such as hormone releasing, feeding behavior, energy balance, and circadian rhythm. Many of these hypothalamic regulatory processes undergo alterations with aging, thereby facilitating the occurrence of age-related diseases.^3^ A recent study utilized single-cell nuclear sequencing to analyze young and aging female mouse hypothalamus, and specific neurons critical for metabolic regulation and body composition showed significant changes in aging hypothalamus.^4^ It is well established that obesity can cause hypothalamic dysfunction, further exacerbating the development of metabolic diseases such as type 2 diabetes and hypogonadotropic hypogonadism.^5^ However, there is currently a lack of sufficient evidence regarding how obesity influences the aging process of hypothalamus.

Neurons constitute the fundamental building blocks of the nervous system. Previous studies have shown that neurons undergoe a progressive decline in functionality with age, resulting in a memory and cognition decline.^6^ Aging is the leading risk factor for neurodegenerative diseases such as Alzheimer’s disease (AD) and Parkinson’s disease.^7^ As post-mitotic cells, senescent neurons, similar to other post-mitotic cells, exhibit senescent features such as increased SA-β-gal activity and enhanced expression of γH2AX and p16, rather than cell-cycle arrest.^8^ Cell senescence shares some common pathways and hallmarks, such as mitochondria and lysosome impairment, chromatin alterations, metabolic changes, and enhanced senescence-related secretory phenotype (SASP).^9^ Furthermore, imbalanced protein homeostasis is also a notable characteristic of cell senescence.^10^ Protein misfolding can disrupt protein homeostasis in neurons, leading to endoplasmic reticulum (ER) stress.^11^ ER stress has been proven to be a risk factor for aging-related neurodegenerative diseases, causing neurodegeneration and neuronal death. ER stress is mediated by three ER transmembrane sensor proteins: activating transcription factor 6 (ATF6), protein kinase RNA-like ER kinase (PERK), and inositol-requiring enzyme 1 (IRE1).^12^ Previous studies have confirmed that the activation of ATF6 promotes fibroblast aging through the COX2/PGE2 pathway. Activation of PERK and IRE1 promotes the generation of SASP.^13^ Additionally, hypothalamic neuronal damage in obesity and aging is, at least in part, attributed to protein imbalance.^14^ Therefore, it can be speculated that alleviating ER stress in hypothalamic neurons may potentially delay hypothalamic aging and improve its function.

A vast body of evidence suggests that obesity is accompanied by chronic systemic inflammation. Moreover, obesity-induced hypothalamic inflammation is thought to be a fundamental cause of hypothalamic dysfunction.^15^ Innovative studies have found that hypothalamic inflammation is associated with the onset of aging.^16^ As inflammation is a standard marker of obesity and aging,^17^ suppressing hypothalamic inflammation may be the key to alleviating the aging process in the hypothalamus of obese patients. Microglia, considered resident immune cells in the CNS, are crucial mediators of the immune response to neuroinflammation.^18^ Obesity activates microglia in the hypothalamus and mediates inflammation following the secretion of pro-inflammatory cytokines and chemokines.^19^ NLRP3 inflammatory vesicles, a well-studied multiprotein complex composed of NLRP3, apoptosis-associated speckle-like protein (ASC), and caspase-1,^20^ trigger a robust pro-inflammatory response upon activation. It has been shown that NLRP3 activation in microglia promotes inflammation in AD.^21^

In addition, NLRP3 activation in a high-fat diet (HFD)-induced obese state is linked with the pathogenesis of brain inflammation.^22^Yet, little is known about the role of NLRP3 inflammatory vesicle activation in the hypothalamus of ob/ob obese mice. Stimulator of interferon genes (STING) is a cytoplasmic DNA sensor widely distributed in mammalian immune cells (e.g., macrophages, microglia, etc.) and is an essential mediator during the regulation of the innate immune response.^23^ Obesity causes activation of the cyclic GMP-AMP synthase (cGAS)/STING pathway, resulting in the development of hippocampal inflammation.^24^ Notably, it has been demonstrated that loss of the mitochondrial inner membrane protein beta carotene oxygenase 2 (BCO2) in the hypothalamus elevates STING protein levels and induces hypothalamic inflammation.^25^ Nevertheless, the role of STING in the hypothalamus of obese mice remains unclear. Interestingly, studies have demonstrated an interaction between STING and NLRP3. Lipopolysaccharide (LPS)-stimulated STING activation triggers NLRP3 activation, which in turn triggers myocardial inflammation, and STING knockdown inhibits NLRP3-mediated inflammation.^26^ Thus, STING and NLRP3 inflammatory vesicle activation inhibition may help ameliorate hypothalamic inflammation and slow down aging.

Metformin is a first-line drug widely used clinically in treating type 2 diabetes.^27^ Furthermore, it has been shown to have anti-inflammatory and anti-aging effects.^28^ Clinical studies have demonstrated that metformin reduces the incidence of many aging-related diseases, such as degenerative bone disease, cardiovascular disease, neurodegenerative disease, and cancer, regardless of whether or not one has diabetes.^29^Previous studies have indicated that metformin prevents the occurrence of ER stress in hepatocytes of obese mice^30^ and has anti-inflammatory effects in various tissues, including adipose, liver, and testis, where it inhibits cellular production of tumor necrosis factor alpha (TNF-α) and interleukin-6 (IL-6) through the activation of adenosine 5‘-monophosphate (AMP)-activated protein kinase (AMPK).^31^ In addition, timely studies have found that in adipose tissue, metformin inhibits inflammation and adipocyte senescence in the obese state by regulating the adipocyte cycle program.^32^ Still, whether metformin alleviates hypothalamic inflammation, controls neuronal ER status, and improves hypothalamic senescence in the obese state is unclear, and its mechanism must be defined.

Hence, we used ob/ob mice and HFD-induced obese mouse model and administered metformin treatment to investigate whether metformin could alleviate hypothalamic aging. We also explored whether hypothalamic neuronal ER stress occurrence and microglia STING and NLRP3-mediated inflammatory response are possible targets of action to provide an essential strategy for treating obesity-induced hypothalamic aging.

## Results

### Metformin alleviates obesity-related metabolic disorders

**Figure 1:**
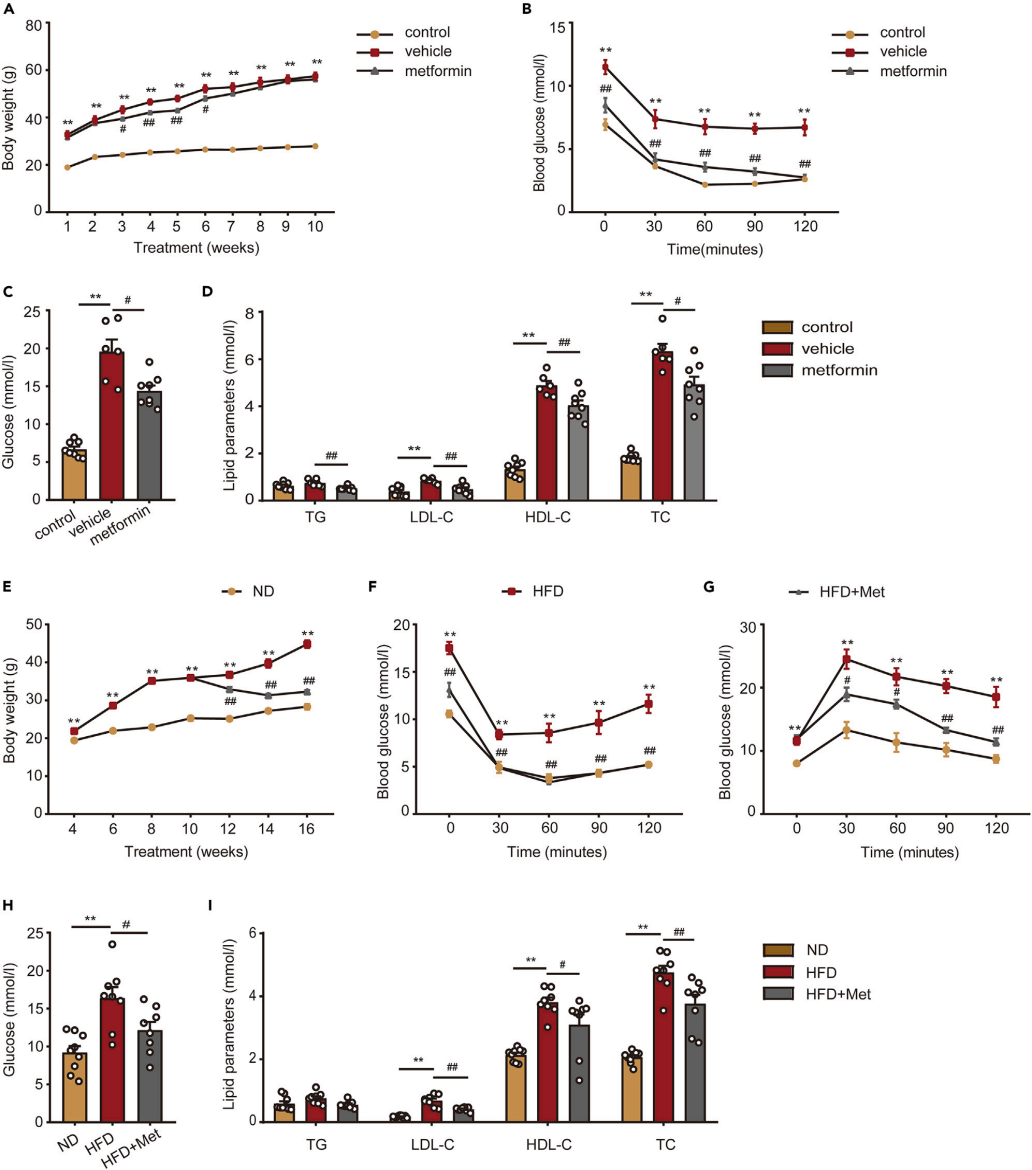
Metformin alleviates obesity-related metabolic disorders (A) Body weight. One-way ANOVA test, ∗∗p < 0.01 vs. control; #p < 0.05, ##p < 0.01 vs. vehicle. The results are shown as the mean ± SEM. (B) ITT. One-way ANOVA test, ∗∗p < 0.01 vs. control; ##p < 0.01 vs. vehicle. The results are shown as the mean ± SEM. (C) fasting blood glucose. One-way ANOVA test, ∗∗p < 0.01 vs. control; #p < 0.05 vs. vehicle. The results are shown as the mean ± SEM. (D) lipid parameters in ob/ob related mice. n = 8 for control, n = 6 for vehicle, n = 8 for metformin. One-way ANOVA test, ∗∗p < 0.01 vs. control; #p < 0.05, ##p < 0.01 vs. vehicle. The results are shown as the mean ± SEM. (E) Body weight. One-way ANOVA test, ∗∗p < 0.01 vs. ND; ##p < 0.01 vs. HFD. The results are shown as the mean ± SEM. (F) ITT. One-way ANOVA test, ∗∗p < 0.01 vs. ND; ##p < 0.01 vs. HFD. The results are shown as the mean ± SEM. (G) GTT. One-way ANOVA test, ∗∗p < 0.01 vs. ND; #p < 0.05, ##p < 0.01 vs. HFD. The results are shown as the mean ± SEM. (H) fasting blood glucose. One-way ANOVA test, ∗∗p < 0.01 vs. ND; #p < 0.05 vs. HFD. The results are shown as the mean ± SEM. (I) lipid parameters in HFD related mice. n = 9 for ND, n = 8 for HFD, n = 8 for HFD+Met. One-way ANOVA test, ∗∗p < 0.01 vs. ND; #p < 0.05, ##p < 0.01 vs. HFD. The results are shown as the mean ± SEM.

First, we measured the body weight of the mice, as seen in Figure 1. The ob/ob mice administrated with saline (vehicle) had significantly higher body weight than C57BL/6 mice (control), although the body weight of mice did not decrease significantly after metformin treatment (metformin) (Figure 1A). To explore the effects of metformin on glucose tolerance and insulin sensitivity in ob/ob mice, we considered fasting blood glucose levels in each group. An insulin tolerance test (ITT) was performed. The experiment showed that metformin improved insulin sensitivity in ob/ob mice and restored it to the level observed in normal mice (Figure 1B). Blood glucose levels were remarkably higher in ob/ob mice compared to control mice, while metformin reduced blood glucose levels (Figure 1C). Next, the effect of metformin on lipid metabolism was assessed by analyzing typical biochemical markers. Our results indicate that serum levels of triglycerides (TGs), total cholesterol (TC), and low-density lipoprotein cholesterol (LDL-C) were significantly higher in ob/ob mice than in control mice. All lipid levels decreased rapidly after metformin treatment; still, they did not return to normal (Figure 1D). Ob/ob mice are an obese model carrying a genetic mutation in the *Leptin* gene, which does not fully represent the models of obesity. Therefore, we also conducted experiments in HFD-induced obese mice. Control mice were fed with HFD for 6 weeks, after which the obese mice were randomly divided into two groups. The normal diet (ND) group and one HFD group received saline as the control groups, while the other HFD group (HFD+Met) received metformin. During the 6-week treatment period, metformin significantly reduced the body weight of HFD mice (Figure 1E) and improved insulin sensitivity and glucose tolerance (Figures 1F and 1G). Similar to the results observed in ob/ob mice, metformin also improved fasting blood glucose (Figure 1H) and lipid metabolism levels (Figure 1I) of HFD-induced obese mice. This suggested that metformin could alleviate the disorder of glucose and lipid metabolism in obese mice.

### Metformin improves hypothalamic aging in obese mice

**Figure 2:**
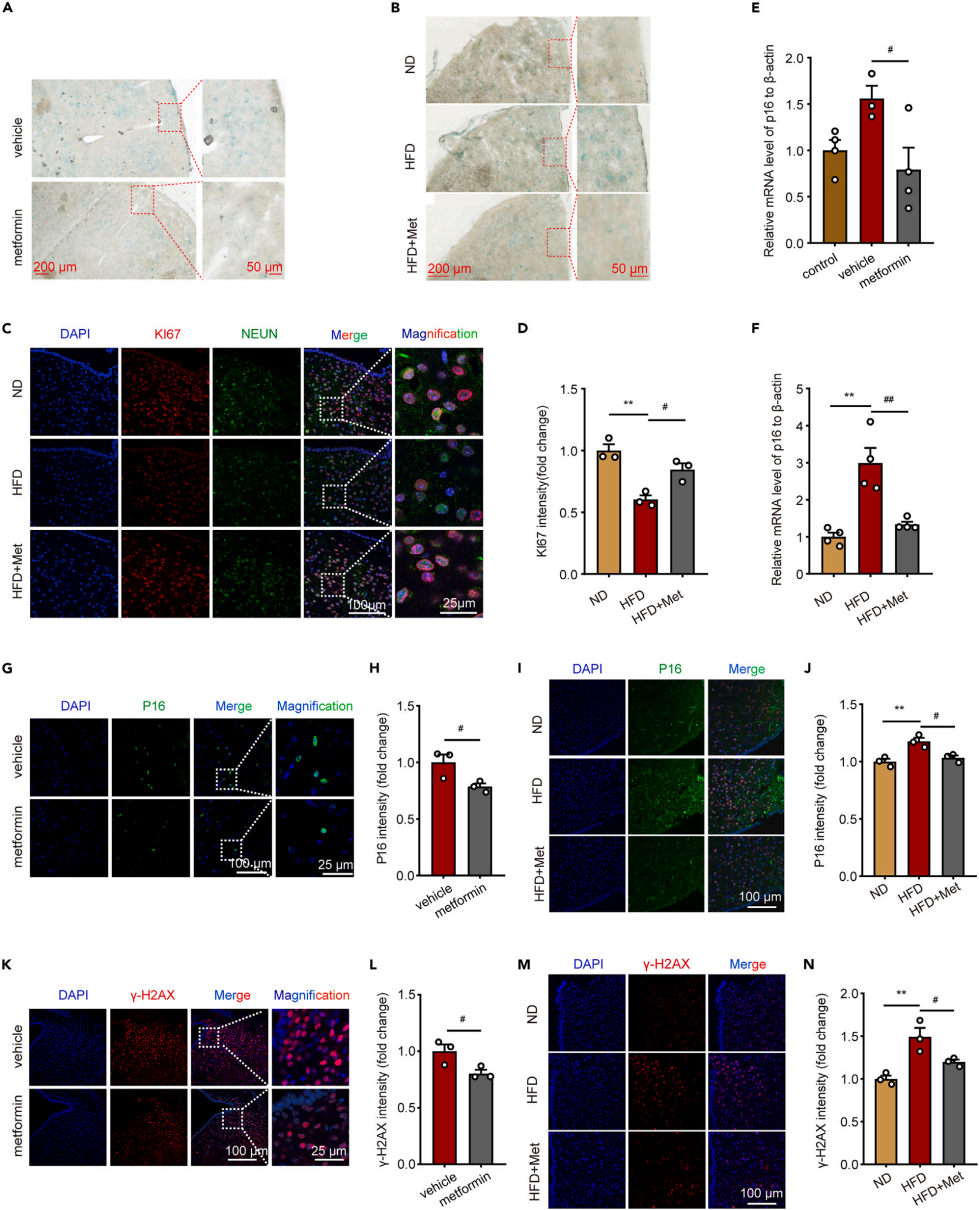
Metformin improves hypothalamic aging in obese mice (A and B) Representative SA-β-gal staining in hypothalamus of ob/ob related mice and HFD-fed related mice. Bar means 50 μm or 200 μm. (C) Representative immunofluorescence staining of KI67 in hypothalamus of HFD-fed related mice. Bar means 25 μm or 100 μm. (D) KI67 intensity in hypothalamus of HFD-fed related mice. n = 3 for each group. One-way ANOVA test, ∗∗p < 0.01 vs. ND; #p < 0.05 vs. HFD. The results are shown as the mean ± SEM. (E and F) The mRNA levels of p16 in hypothalamus of ob/ob related mice and HFD-fed related mice. n = 3 for vehicle, n = 4 for other groups. One-way ANOVA test, ∗∗p < 0.01 vs. ND; #p < 0.05, ##p < 0.01 vs. vehicle or HFD. The results are shown as the mean ± SEM. (G and I) Representative immunofluorescence staining of P16 in hypothalamus of ob/ob related mice and HFD-fed related mice. Bar means 25 μm or 100 μm. (H and J) P16 intensity in hypothalamus of ob/ob related mice and HFD-fed related mice. n = 3 for each group. An unpaired two-tailed t test between two groups, one-way ANOVA test among three groups, ∗∗p < 0.01 vs. ND; #p < 0.05, vs. vehicle or HFD. The results are shown as the mean ± SEM. (K and M) Representative immunofluorescence staining of γ-H2AX in hypothalamus of ob/ob related mice and HFD-fed related mice. Bar means 25 μm or 100 μm. (L and N) γ-H2AX intensity in hypothalamus of ob/ob related mice and HFD-fed related mice. n = 3 for each group. An unpaired two-tailed t test between two groups, one-way ANOVA test among three groups, ∗∗p < 0.01 vs. ND; #p < 0.05, vs. vehicle or HFD. The results are shown as the mean ± SEM.

Further assessment of hypothalamic tissue senescence and the effect of metformin was performed on obese mice. As shown in Figures 2A and 2B, SA-β-gal staining was reduced in the metformin-treated group relative to the hypothalamus of both ob/ob mice and HFD-fed mice. In addition, RT-PCR was performed to detect the expression of cellular senescence-related marker p16. The results indicate that p16 expression levels were increased in the hypothalamus of both ob/ob mice and HFD-fed mice and returned to normal levels following metformin treatment (Figures 2E and 2F). The arcuate nucleus (ARC) region of the hypothalamus plays a critical role in sensing metabolic signals in the periphery and regulating energy intake and expenditure. Subsequent analysis of P16 and γ-H2AX in the ARC region utilizing immunofluorescence techniques revealed co-staining of P16 and γ-H2AX with NeuN, a specific neuronal marker, in obese mice. In contrast, P16 did not exhibit co-staining with IBA1, a specific microglial marker, suggesting that obesity promoted neuronal senescence but not in microglia (Figure S1). Importantly, the administration of metformin significantly downregulated the expression of both P16 and γ-H2AX (Figures 2G–2N), indicating its potential to mitigate neuronal senescence. Furthermore, we also conducted TUNEL staining and KI67 staining to examine cellular apoptosis and proliferation. TUNEL staining revealed that metformin exhibited no effects on neuronal apoptosis, both *in vivo* and *in vitro* (Figure S2). Our findings demonstrate a reduction in KI67 staining intensity in neuronal cells upon palmitic acid (PA) stimulation *in vitro*. Treatment with metformin was able to rescue the diminished expression of KIi67 (Figures 2C and 2D). These results are consistent with previous studies indicating that metformin promotes neuronal proliferation in obese mice. Based on these results, obesity induces the aging of neurons in the ARC region of the hypothalamus, whereas metformin has an inhibitory effect on aging.

### Metformin alleviates ER stress in hypothalamic neurons of obese mice

**Figure 3:**
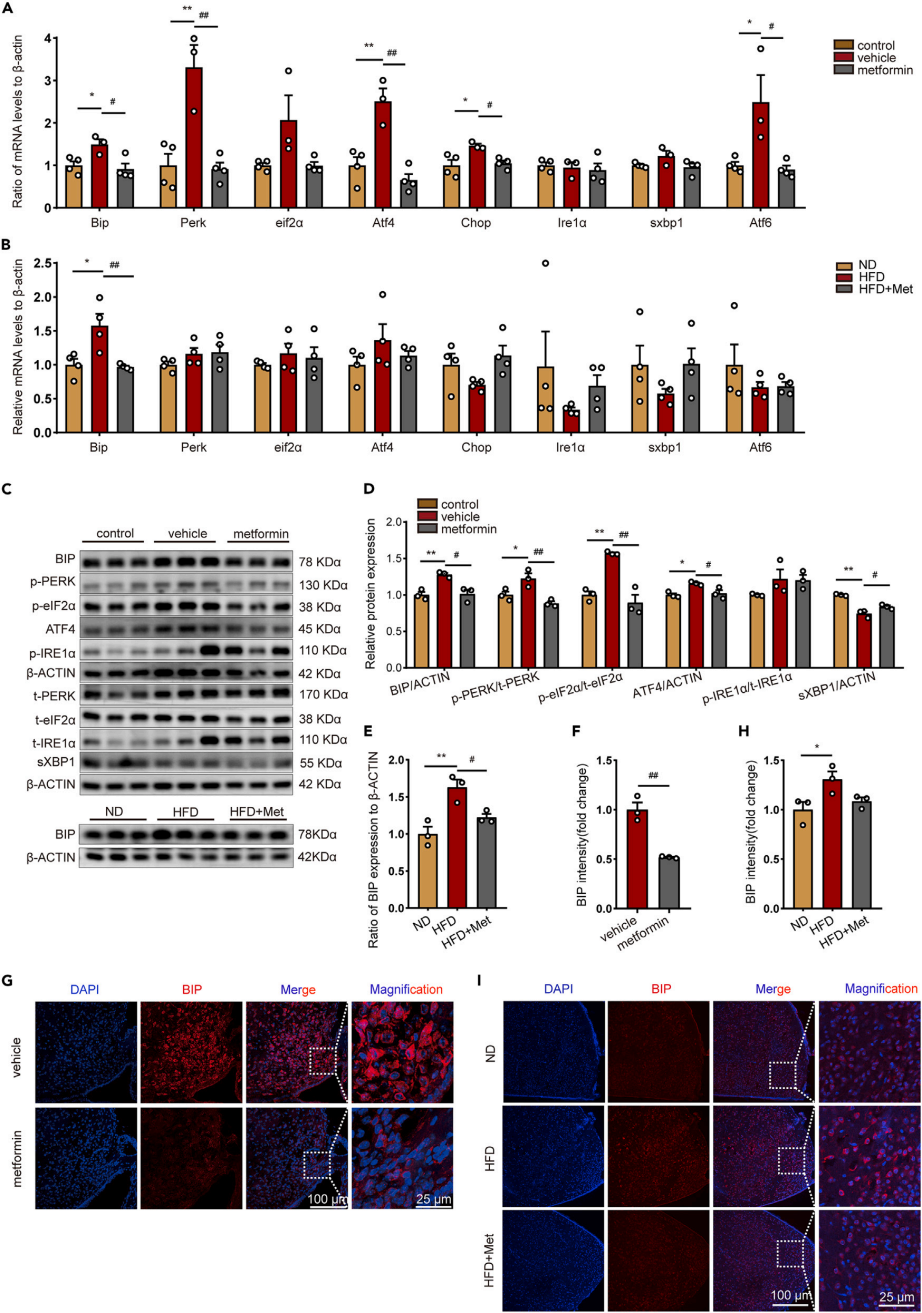
Metformin alleviates ER stress in hypothalamic neurons of obese mice (A and B) The mRNA levels of Bip, Perk, eif2α, Atf4, Chop, Ire1α, sxbp1, and Atf6 in hypothalamus of ob/ob related mice and HFD-fed related mice. n = 3 for vehicle, n = 4 for other groups. One-way ANOVA test, ∗p < 0.05, ∗∗p < 0.01 vs. control or ND; #p < 0.05, ##p < 0.01 vs. vehicle or HFD. The results are shown as the mean ± SEM. (C) Representative immunoblotting of BIP, PERK, eIF2α, ATF4, IRE1α, and sXBP1 in hypothalamus of ob/ob related mice and HFD-fed related mice. (D and E) The protein levels of BIP, PERK, eIF2α, ATF4, IRE1α, and sXBP1 in hypothalamus of ob/ob related mice and HFD mice. n = 3 for each group. One-way ANOVA test, ∗p < 0.05, ∗∗p < 0.01 vs. control or ND; #p < 0.05, ##p < 0.01 vs. vehicle or HFD. The results are shown as the mean ± SEM. (F and H) BIP intensity in hypothalamus of ob/ob related mice and HFD-fed related mice. n = 3 for each group. An unpaired two-tailed t test between two groups, one-way ANOVA test among three groups, ∗p < 0.05 vs. ND; ##p < 0.01 vs. vehicle. The results are shown as the mean ± SEM. (G and I) Representative immunofluorescence staining of BIP in hypothalamus of ob/ob related mice and HFD-fed related mice. Bar means 25 μm or 100 μm.

ER stress is linked with aging and neurodegenerative diseases. Therefore, ER stress biomarker expression and phosphorylation levels were further examined, assessing the level of ER stress in the hypothalamus of obese mice and the specific effect of metformin on it. According to RT-PCR experiments, *Leptin* deficiency significantly upregulated the hypothalamic immunoglobulin heavy chain binding protein (Bip), PKR-like ER kinase (Perk), eukaryotic translation initiation factor 2 subunit alpha (eif2α), activating transcription factor 4 (Atf4), C/EBP Homologous Protein (Chop), and activating transcription factor 6 (Atf6) expression at the mRNA level. At the same time, metformin lowered the expression levels of these genes and largely restored them to the level of normal mice (Figure 3A). HFD induced a significant increase in Bip expression in the hypothalamus, and metformin treatment was observed to mitigate this increase, whereas other ER stress markers showed few changes (Figure 3B). After further analysis of their protein expression levels, it was clear that phosphorylation and protein expression levels of BIP and PERK-related pathways, including PERK, eIF2α, and ATF4, were sharply increased in ob/ob mice compared with control mice, and phosphorylation levels and protein expression were significantly lower after metformin treatment (Figures 3C and 3D). In contrast, no notable effects were observed regarding phosphorylation levels and the protein expression of IRE1α-related pathways, including IRE1α (Figures 3C and 3D). We further assessed the protein levels of BIP in the hypothalamus of HFD-fed mice. As depicted in Figures 3C and 3E, HFD led to an upregulation of BIP expression, whereas treatment with metformin attenuated this increase in BIP levels. Moreover, immunofluorescence further discovered that BIP, an essential regulator of ER stress, was enormously increased in the hypothalamic ARC region in the ob/ob group, and metformin treatment decreased its expression (Figures 3F and 3G). Similar results were observed in the hypothalamic ARC region of HFD-fed mice (Figures 3H and 3I). These results suggest that obese mice activated ER stress and PERK-related unfolded protein response pathways in the hypothalamus and that metformin was effective in alleviating ER stress.

### Metformin ameliorates hypothalamic inflammation in obese mice

**Figure 4:**
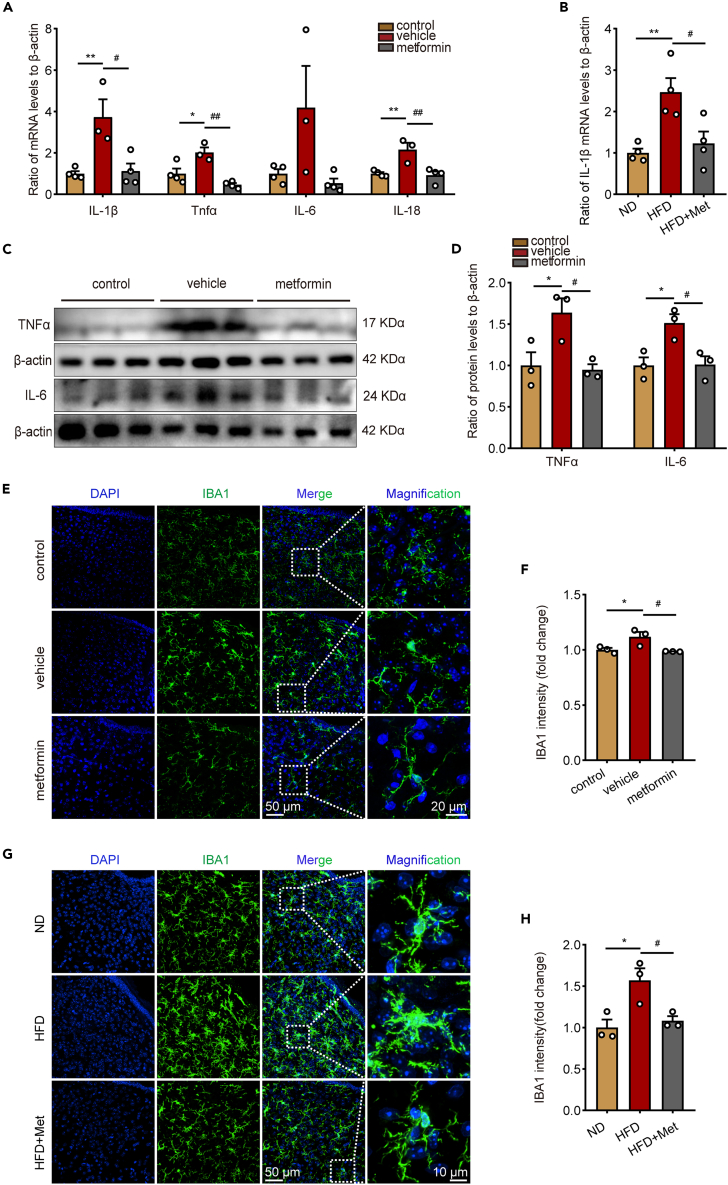
Metformin ameliorates hypothalamic inflammation in obese mice (A) The mRNA levels of Il-1β, Tnf-α, Il-6, and Il-18 in hypothalamus of ob/ob related mice. n = 3 for vehicle, n = 4 for control and metformin group. One-way ANOVA test, ∗p < 0.05, ∗∗p < 0.01 vs. control; #p < 0.05, ##p < 0.01 vs. vehicle. The results are shown as the mean ± SEM. (B) The mRNA levels of Il-1β in hypothalamus of HFD-fed related mice. n = 4 for each groups. One-way ANOVA test, ∗∗p < 0.01 vs. ND; #p < 0.05 vs. HFD. The results are shown as the mean ± SEM. (C) Representative immunoblotting of TNF-α and IL-6 in hypothalamus of ob/ob related mice. (D) The protein levels of TNF-α and IL-6 in hypothalamus of ob/ob related mice. One-way ANOVA test, ∗p < 0.05 vs. control; #p < 0.05 vs. vehicle. The results are shown as the mean ± SEM. (E and G) Representative immunofluorescence staining of IBA1 in hypothalamus of ob/ob related mice and HFD-fed related mice. Bar means 10 μm, 20 μm or 50 μm. (F and H) IBA1 intensity in hypothalamus of ob/ob related mice and HFD-fed related mice. n = 3 for each group. One-way ANOVA test, ∗p < 0.05 vs. control or ND; #p < 0.05 vs. vehicle or HFD. The results are shown as the mean ± SEM.

Notably, hypothalamic inflammation is found to be the key to aging. To further investigate if metformin ameliorated hypothalamic inflammation in obese mice, the levels of pro-inflammatory cytokines in the hypothalamus were considered. According to the results, compared with control mice, hypothalamic pro-inflammatory cytokines were sharply increased in ob/ob mice at the mRNA level, including Tnfα, Il-1β, Il-6, and Il-18, and metformin could mitigate the upregulation of these pro-inflammatory cytokines (Figure 4A). The expression of Il-1β was also decreased by metformin in HFD-fed mice (Figure 4B). In addition, TNFα and IL-6 were also significantly increased at the protein level, while metformin treatment effectively lowered the mRNA and protein expression levels of pro-inflammatory cytokines in ob/ob mice (Figures 4C and 4D). Activated microglia are a significant source of pro-inflammatory cytokines and crucial mediators of the hypothalamic inflammatory response. Obesity-induced hypothalamic inflammation occurs primarily in the ARC region.^33^ Next, we analyzed the effect of metformin on IBA1-positive microglia in the ARC area by detecting their number and morphology by utilizing immunofluorescence techniques. The results indicate that microglia in the ARC area of the hypothalamus were lowered in number in the metformin-administered group and showed longer branches and smaller cell bodies both in ob/ob mice (Figures 4E and 4F) and HFD-fed mice (Figures 4G and 4H). These findings highlight how metformin attenuates the hypothalamic inflammatory response and inhibits microglia activation in obese mice.

### Metformin inhibits the activation of STING and NLRP3 in hypothalamic microglia of obese mice

**Figure 5:**
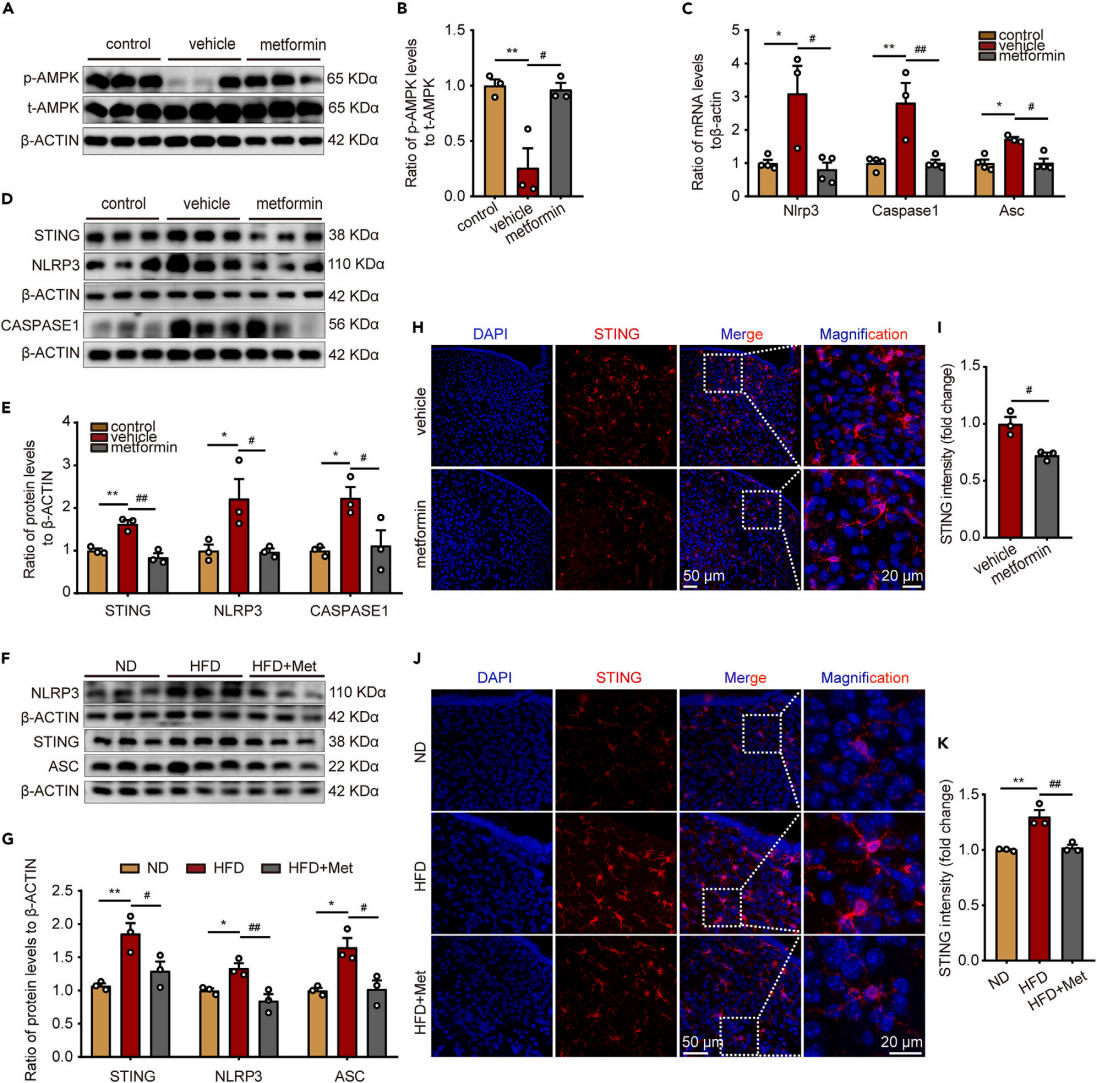
Metformin inhibits the activation of STING and NLRP3 in hypothalamic microglia of obese mice (A) Representative immunoblotting of AMPK in hypothalamus of ob/ob related mice. (B) The protein levels of *p*-AMPK/t-AMPK in hypothalamus of ob/ob related mice. n = 3 for each group. One-way ANOVA test, ∗∗p < 0.01 vs. control; #p < 0.05 vs. vehicle. The results are shown as the mean ± SEM. (C) The mRNA levels of Nlrp3, Caspase1, and Asc in hypothalamus of ob/ob related mice. n = 3 for vehicle, n = 4 for control and metformin group. One-way ANOVA test, ∗p < 0.05, ∗∗p < 0.01 vs. control; #p < 0.05, ##p < 0.01 vs. vehicle. The results are shown as the mean ± SEM. (D and F) Representative immunoblotting of STING, NLRP3, CASPASE1, and ASC in hypothalamus of ob/ob related mice and HFD-fed related mice. (E and G) The protein levels of STING, NLRP3, CASPASE1, and ASC in hypothalamus of ob/ob related mice and HFD-fed related mice. n = 3 for each group. One-way ANOVA test, ∗p < 0.05, ∗∗p < 0.01 vs. control or ND; #p < 0.05, ##p < 0.01 vs. vehicle or HFD. The results are shown as the mean ± SEM. (H and J) Representative immunofluorescence staining of STING in hypothalamus of ob/ob related mice and HFD-fed related mice. Bar means 20 μm or 50 μm. (I and K) STING intensity in hypothalamus of ob/ob related mice and HFD-fed related mice. N = 3 for each group. An unpaired two-tailed t test between two groups, one-way ANOVA test among three groups, ∗∗p < 0.01 vs. ND; #p < 0.05, ##p < 0.01 vs. vehicle or HFD. The results are shown as the mean ± SEM.

According to previous studies, hypothalamic AMPK is associated with energy metabolic pathways like feeding and thermogenesis. AMPK is a direct target molecule of metformin, which can exert its effects by regulating AMPK phosphorylation levels.^34^ For further assessment of the role of metformin in the hypothalamus, protein blotting was used to analyze the expression of AMPK-related proteins in the hypothalamus. Notably, AMPK phosphorylation levels were significantly reduced in the hypothalamus of ob/ob mice compared to C57BL/6 mice and vastly increased after metformin treatment (Figures 5A and 5B). Since STING and NLRP3 inflammatory vesicles play an essential role in the development of inflammation, their changes in the hypothalamus and the effect of metformin on them were further explored in depth. The results indicated that metformin lowered the expression of Nlrp3, Caspase1, and Asc at the mRNA level (Figure 5C). STING, NLRP3, and CASPASE1 protein levels were distinctly elevated in the hypothalamus of ob/ob mice compared to C57BL/6 mice, and metformin treatment downregulated these proteins’ expression (Figures 5D and 5E). We additionally investigated the protein expression levels of STING, NLRP3, and ASC within the hypothalamus of mice fed with HFD. Metformin treatment effectively mitigated the HFD-induced upregulation of these proteins, as illustrated in Figures 5F and 5G. Analysis of STING expression in the ARC region utilizing immunofluorescence outlined how STING expression was strongly downregulated in microglia after metformin treatment compared to ob/ob mice and HFD-fed mice (Figures 5H–5K). Based on these results, metformin may be involved in regulating inflammation by promoting AMPK phosphorylation and inhibiting the activation of STING and NLRP3 inflammatory vesicles in the ARC region of the hypothalamus in obese mice.

### Metformin alleviates neuronal senescence by suppressing microglia inflammation *in vitro*

**Figure 6:**
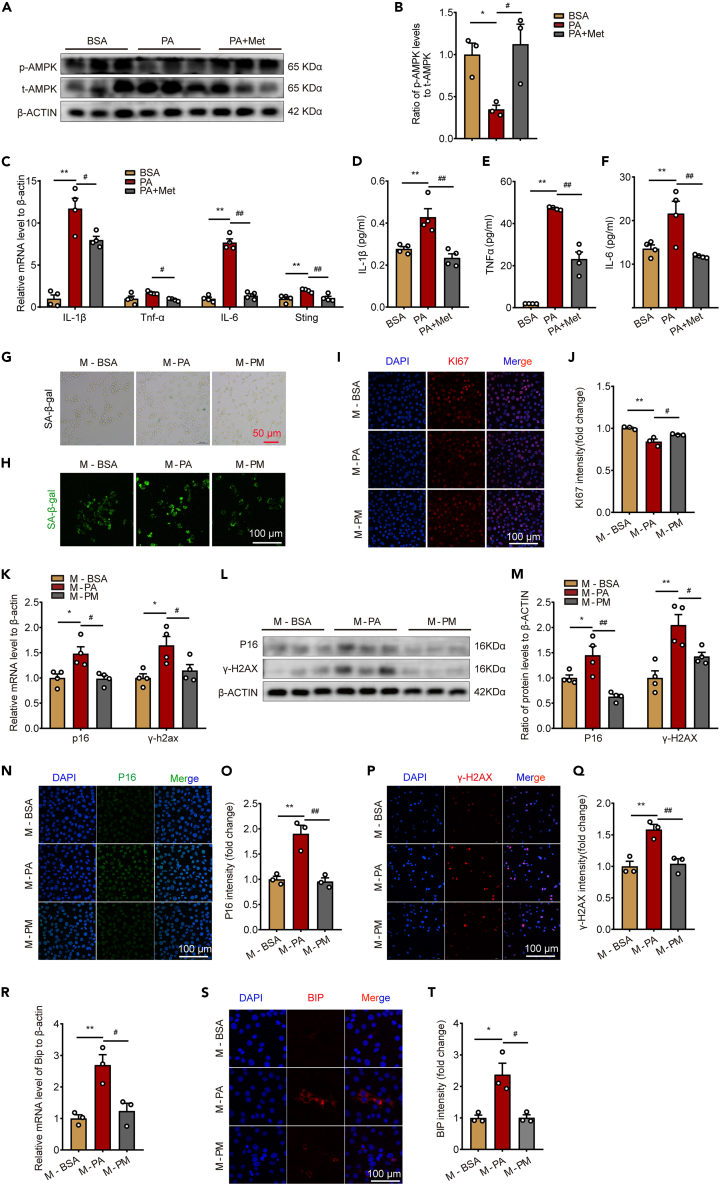
Metformin alleviates neuronal senescence by suppressing microglia inflammation *in vitro* (A) Representative immunoblotting of AMPK in BV2 cells. (B) The protein levels of *p*-AMPK/t-AMPK in BV2 cells. N = 3 for each group. One-way ANOVA test, ∗p < 0.05 vs. BSA; #p < 0.05 vs. PA. The results are shown as the mean ± SEM. (C) The mRNA levels of Il-1β, Tnf-α, Il-6, and Sting in BV2 cells. n = 3 for each group. One-way ANOVA test, ∗∗p < 0.01 vs. BSA; #p < 0.05, ##p < 0.01 vs. PA. The results are shown as the mean ± SEM. (D–F) The levels of IL-1β, TNFα, and IL-6 in the culture supernatant of BV2 cells. One-way ANOVA test, ∗∗p < 0.01 vs. BSA; ##p < 0.01 vs. PA. The results are shown as the mean ± SEM. (G and H) Representative SA-β-gal staining and immunofluorescence staining in POMC neurons. Bar means 50 μm or 100 μm. (I) Representative immunofluorescence staining of KI67 in POMC neurons. Bar means 100 μm. (J) KI67 intensity in POMC neurons. n = 3 for each group. One-way ANOVA test, ∗∗p < 0.01 vs. M-BSA; #p < 0.05 vs. M-PA. The results are shown as the mean ± SEM. (K) The mRNA levels of p16 and γ-H2AX in POMC neurons. n = 4 for each group. One-way ANOVA test, ∗p < 0.05 vs. M-BSA; #p < 0.05 vs. M-PA. The results are shown as the mean ± SEM. (L) Representative immunoblotting of P16 and γ-H2AX in POMC neurons. (M) The protein levels of P16 and γ-H2AX in POMC neurons. n = 4 for each group. One-way ANOVA test, ∗p < 0.05, ∗∗p < 0.01 vs. M-BSA; #p < 0.05, ##p < 0.01 vs. M-PA. The results are shown as the mean ± SEM. (N and P) Representative immunofluorescence staining of P16 and γ-H2AX in POMC neurons. Bar means 100 μm. (O and Q) P16 and γ-H2AX intensity in POMC neurons. n = 3 for each group. One-way ANOVA test, ∗∗p < 0.01 vs. M-BSA; ##p < 0.01 vs. M-PA. The results are shown as the mean ± SEM. (R) The mRNA levels of Bip in POMC neurons. n = 3 for each group. One-way ANOVA test, ∗∗p < 0.01 vs. M-BSA; #p < 0.05 vs. M-PA. The results are shown as the mean ± SEM. (S) Representative immunofluorescence staining of BIP in POMC neurons. Bar means 100 μm. (T) BIP intensity in POMC neurons. n = 3 for each group. One-way ANOVA test, ∗p < 0.05 vs. M-BSA; #p < 0.05 vs. M-PA. The results are shown as the mean ± SEM.

To further investigate whether metformin improves neuronal senescence by modulating neuroinflammation, we first cultured BV2 microglia cell line *in vitro* and treated with PA and metformin. Metformin effectively restored the suppressed phosphorylation of AMPK caused by PA stimulation (Figures 6A and 6B). Moreover, the upregulation of Il-1β, Tnf-α, Il-6, and Sting mRNA levels in the cells induced by PA was effectively reversed by metformin (Figure 6C). Additionally, metformin demonstrated a significant reduction in the levels of IL-1β, TNFα, and IL-6 in the cell culture supernatant, which were elevated due to PA stimulation (Figures 6D–6F). These findings suggest that metformin exerts potent anti-inflammatory effects in microglia. Subsequently, we collected the supernatants from each group to treat pro-opiomelanocortin (POMC) neuronal cells. The results demonstrated that the metformin-treated group significantly suppressed the PA-induced increase in SA-β-gal activity and expression (Figures 6G and 6H), as well as reversed the decrease in KI67 expression caused by PA (Figures 6I and 6J). Additionally, we assessed the expression levels of the senescence markers p16 and γ-H2AX in each group. Consistent with the *in vivo* findings, both the mRNA and protein levels of p16 and γ-H2AX were significantly decreased in the metformin-treated group (Figures 6K–Q). Moreover, the expression of ER stress-related protein BIP was also significantly inhibited by metformin (Figures 6R–T). These findings indicate that metformin prominently inhibits the expression of microglia-derived cytokines, and neuroinflammation may partially mediate the anti-neuronal senescence effects of metformin.

## Discussion

An extensive body of evidence suggests that obesity is closely related to aging. As a critical brain region regulating reproduction, endocrine energy metabolism, and other functions, the hypothalamus plays a vital role in vertebrates' aging process.^35^ Nevertheless, the effects and mechanisms of obesity on hypothalamic aging lack detailed studies. Metformin has been proven to have anti-inflammatory and anti-aging effects in various tissues, but its ability to ameliorate hypothalamic inflammation and delay aging remains unclear. In the present study, we discovered that metformin significantly improved abnormal lipids, glucose, and insulin sensitivity in ob/ob mice, confirming its future therapeutic potential for metabolic syndrome. The subsequent studies demonstrated that metformin delayed the hypothalamic aging process in obese mice, which can act by inhibiting hypothalamic inflammation triggered by activation of ER stress in neurons and STING and NLRP3 inflammatory vesicles in microglia.

The inability of ob/ob mice to synthesize leptin leads the organism to exhibit a state of morbid obesity and hyperphagia.^36^ In this study, we found that metformin treatment was ineffective in improving body weight gain compared to ob/ob mice not given metformin, consistent with the results of 1,3-butanediol and celastrol treatment of body weight in ob/ob mice,^37^^,^^38^while metformin treatment could significantly reduce the body weight of HFD-fed mice. Metformin has been reported as a *Leptin* sensitizer, enhancing the production of leptin receptors, which may contribute to improved leptin sensitivity and the reversal of obesity.^39^ However, in ob/ob mice, which exhibit obesity due to the inactivation mutation of the leptin gene, metformin may not exert its leptin-sensitizing effects through promoting leptin receptor expression, resulting in minimal or negligible effects on body weight regulation. Studies have highlighted that obesity prevails as a risk factor for metabolic syndrome and the basis for developing insulin resistance (IR). We evaluated whether metformin could improve IR in ob/ob mice and HFD-fed mice. It was found that after metformin treatment, blood glucose levels were remarkably lower, indicating that metformin improved insulin sensitivity just as previously reported. Obesity-induced dyslipidemia is manifested by higher TC, TG, and LDL-C levels.^40^We further analyzed the serum lipid levels in obese mice treated with metformin. The blood levels of TG, TC, and LDL-C in metformin-treated mice were lower than those in the obese untreated group, even though they were still higher than those in normal mice. These results were in line with those of Wang et al.,^41^ suggesting that metformin treatment can improve obesity-induced disturbances in glucose and lipid metabolism and that this effect does not depend on weight loss. It is intriguing that both ob/ob mice and HFD-fed mice exhibit elevated levels of serum high-density lipoprotein cholesterol (HDL-c), which appears to contradict the conclusion of several population studies that have reported a negative correlation between adiposity and HDL-c levels.^42^ However, it is worth noting that other studies have also reported an increase in serum HDL-c levels in obese mice.^43^ One possible explanation is that dietary fat increases the transport rates and decreases the fractional catabolic rates of HDL-cholesterol ester and apo A-I.^44^ Additionally, Tall et al. previously reported that obese (ob/ob) mice exhibit impaired HDL binding, uptake, and recirculation, leading to increased plasma HDL levels.^45^ Therefore, the elevated HDL levels observed in ob/ob mice and HFD-fed mice may have different underlying reasons.

Obesity leads to an increase in aging, associated with changes such as proteostasis imbalance, nutrient sensing dysregulation, mitochondrial dysfunction, cellular senescence, and chronic inflammation.^46^ The hypothalamus is a critical brain region that regulates endocrine and metabolism; on the one hand, obesity may promote hypothalamic aging and lead to hypothalamic dysfunction. On the other hand, hypothalamic dysfunction may result in metabolic disorders and accelerate organismal aging. Prior held studies have reported that metformin can treat aging-related cognitive dysfunction.^47^ Cellular senescence is the basis of organ aging, manifested by permanent cell-cycle arrest regulated by p16 and p21.^48^ Studies have indicated that DNA damage is a major cause of cellular senescence.^48^ Furthermore, the DNA damage marker γ-H2AX was significantly higher in HFD-induced rat brain tissue.^49^In our study, hypothalamic p16 and γ-H2AX were increased at the gene and protein levels in obese mice, and metformin lowered the extent of this change. Hence, it is suggested that obesity may damage DNA, leading to cell-cycle arrest and triggering hypothalamic senescence. At the same time, metformin can alleviate cellular senescence by delaying DNA damage, thus ameliorating hypothalamic senescence.

Studies have indicated that protein homeostasis networks and ER stress are essential regulators of aging. Park et al. demonstrated that the ER stress marker BIP was enormously elevated in hypothalamic neurons of 6–8 weeks adult ob/ob mice.^50^ Our study has shown that both BIP and PERK-related pathways were activated, and gene expression levels of CHOP were sharply increased, possibly related to the different ages of the mice, causing increased protein accumulation. Lenox et al. discovered that phosphorylation of CHOP, ATF4, and eIF2α was upregulated in the retina of aged rats.^51^ In line with these findings, our results demonstrate that phosphorylation of these proteins is linked with hypothalamic aging in obese mice, highlighting the vital role of the PERK-eIF2α-ATF4 pathway in aging-related ER stress. Furthermore, we found that in obesity-induced hypothalamic senescence, BIP, an essential molecular chaperone of the ER, is predominantly located in neurons. These phenomena assert the importance of hypothalamic protein homeostasis in obesity and aging. Metformin downregulates the expression in ER stress-related markers, suggesting that it can correct abnormal hypothalamic protein deposition in obese mice.

It is widely known that obesity is associated with hypothalamic inflammation, which is closely related to aging. Studies have proven that obesity disrupts the integrity of the blood-brain barrier (BBB), culminating in its dysfunction, which allows peripheral cytokines and macrophages to enter the hypothalamus and trigger inflammation.^15^ Along with peripheral effects, most studies agree that hypothalamic microglia play a crucial role in inflammation. Diet-induced obesity can trigger microglial activation and production of pro-inflammatory cytokines, developing hypothalamic inflammation. Notably, metformin can act through the BBB and thus in the brain,^52^ a process found to be a prerequisite for metformin inhibition of hypothalamic inflammation. In this study, inflammatory markers like TNF-α, IL-1β, IL-6, and IL-18 were significantly increased in the hypothalamic tissue of ob/ob mice compared to control mice at both transcriptional and protein levels. Simultaneously, metformin treatment inhibited the expression of inflammatory factors. Compared to untreated ob/ob mice, metformin treatment lowered the microglia in the arc region, a region of high hypothalamic inflammation. It inhibited their activation, and results were similar to those reported by Li et al. that metformin downregulates microglia necrotic apoptosis to attenuate hypothalamic inflammation in HFD-induced obesity,^53^ suggesting that the protective effect of metformin on the hypothalamus is partially mediated by the inhibition of microglia-mediated inflammatory responses. Moreover, there is clear evidence that there exists a feedback loop between ER stress and neuroinflammation, a process that is strongly associated with aging-related neurodegenerative diseases and AD,^54^ suggesting that metformin may reduce hypothalamic inflammation, slowing down the aging process by inhibiting hypothalamic microglia activation and alleviating neuronal ER stress.

AMPK is a critical protein for regulating hypothalamic metabolic function and a significant target for the action of metformin. Additionally, AMPK has been shown to regulate several age-related signaling pathways, like SIRT1, nuclear factor κB (NF-κB), and P53, which regulate cellular senescence in mammals.^55^ According to our results, the phosphorylation level of hypothalamic AMPK was remarkably downregulated in obese mice, and its phosphorylation level was upregulated after metformin treatment, suggesting that AMPK may be the leading target of metformin involved in mediating hypothalamic aging and metabolism. Current studies have reported that STING and NLRP3 inflammatory vesicle activation in microglia has been crucial in neuroinflammation.^56^ Hence, we confirmed that STING and NLRP3 inflammatory vesicles are aberrantly activated in the hypothalamus of obese mice; however, they are reduced after metformin treatment. Since AMPK activation inhibits STING-induced inflammatory injury in a subarachnoid hemorrhage (SAH) model,^57^ we hypothesized that metformin inhibits hypothalamic inflammation following the AMPK-STING pathway. Furthermore, the STING pathway affects neuroinflammation, DNA damage, and senescence, and metformin can inhibit myeloid senescence by modulating the STING pathway.^58^ As such, it is proposed that metformin may inhibit hypothalamic inflammation through the AMPK-STING pathway, alleviating hypothalamic senescence, implying an innovative application of metformin in the treatment of STING pathway-related hypothalamic senescence.

### Limitations of the study

In summary, our study shows how metformin delays obesity-induced hypothalamic aging (schematic diagram). Reduced hypothalamic microglia activation, pro-inflammatory cytokine levels, and neuronal ER can lead to this effect. A deeper exploration of the mechanism may depend on the activation of AMPK and inhibition of STING and NLRP3 inflammatory vesicles activation in microglia. The interconnected network between aging, microglia cell-induced inflammation, and neuronal ER stress deserves further research in future studies.

## STAR★Methods

### Key resources table

**Table:** 

REAGENT or RESOURCE	SOURCE	IDENTIFIER
**Antibodies**		

Rabbit polyclonal anti-NLRP3	Novus	Cat# NBP2-12446; RRID: AB_2750946
Mouse Monoclonal anti-Caspase1	Novus	Cat# NB100-56565; RRID: AB_837823
Rabbit Monoclonal anti-ASC	Cell Signaling Technology	Cat#67824; RRID: AB_2799736
Rabbit polyclonal anti-STING	Proteintech	Cat#19851-1-AP; RRID: AB_10665370
Rabbit polyclonal anti-KI67	Proteintech	Cat#27309-1-AP; RRID: AB_2756525
Mouse Monoclonal anti-IL-6	Proteintech	Cat#66146-1-Ig; RRID: AB_2881543
Rabbit polyclonal anti-TNF-α	Abcam	Cat# ab6671; RRID: AB_305641
Rabbit polyclonal anti-BIP	Proteintech	Cat#11587-1-AP; RRID: AB_2119855
Rabbit polyclonal anti-ATF4	Proteintech	Cat#10835-1-AP; RRID: AB_2058600
Rabbit Monoclonal anti-IRE1α (phospho S724)	Abcam	Cat# ab124945; RRID: AB_11001365
Rabbit polyclonal anti-IRE1α	Abcam	Cat# ab37073; RRID: AB_775780
Rabbit Monoclonal anti-p-PERK (Thr980)	Cell Signaling Technology	Cat#3179; RRID: AB_2095853
Rabbit Monoclonal anti-PERK	Cell Signaling Technology	Cat#3192; RRID: AB_2095847
Rabbit Monoclonal anti-eIF2α, phospho (Ser51)	Cell Signaling Technology	Cat#3597; RRID: AB_390740
Rabbit polyclonal anti-eIF2α	Cell Signaling Technology	Cat#9722; RRID: AB_2230924
Rabbit polyclonal anti-XBP-1s	Cell Signaling Technology	Cat#83418; RRID: AB_2800016
Rabbit Monoclonal anti-AMPK-α, phospho (Thr172)	Cell Signaling Technology	Cat#2535; RRID: AB_331250
Rabbit polyclonal anti-AMPK-α	Cell Signaling Technology	Cat#2532; RRID: AB_330331
Mouse Monoclonal anti-p16	Santa Cruz Biotechnology	Cat# sc-1661; RRID: AB_628067
Rabbit Monoclonal anti-Histone H2A.X, phospho (Ser139)	Abcam	Cat# ab81299; RRID: AB_1640564
Rabbit Monoclonal anti-NeuN	Abcam	Cat# ab177487; RRID: AB_2532109
Mouse Monoclonal anti-NeuN	Proteintech	Cat#66836-1-Ig; RRID: AB_2882179
Goat polyclonal anti-Iba1	Abcam	Cat# ab5076; RRID: AB_2224402
Mouse Monoclonal anti-β-ACTIN	Proteintech	Cat#66009-1-Ig; RRID: AB_2687938
Donkey anti-rabbit IgG (H + L) Highly Cross-Adsorbed Secondary Antibody, Alexa Fluor^TM^ 555	Thermo Fisher Scientific	Cat#A-31572; RRID: AB_162543
Donkey anti-mouse IgG (H + L) Highly Cross-Adsorbed Secondary Antibody, Alexa Fluor^TM^ 488	Thermo Fisher Scientific	Cat#A-21202; RRID: AB_141607
Donkey anti-mouse IgG (H + L) Highly Cross-Adsorbed Secondary Antibody, Alexa Fluor^TM^ 555	Thermo Fisher Scientific	Cat#A-31570; RRID: AB_2536180
Donkey anti-goat IgG (H + L) Highly Cross-Adsorbed Secondary Antibody, Alexa Fluor^TM^ Plus 488	Thermo Fisher Scientific	Cat# A32814; RRID: AB_2762838

**Chemicals, peptides, and recombinant proteins**		

Metformin	MERCK	CAS:1115-70-4
Palmitic acid	Sigma	Cat#P5585
Phosphatase inhibitor cocktail	Bimake	Cat#B15001
SPiDER-βGal	Dojindo	Cat# SG02
TRIzol reagent	Vazyme, (Nanjing, China)	Cat#R401-01

**Critical commercial assays**		

SA-β-gal staining kit	Beyotime	Cat#C0602
One Step PrimeScript® RT Reagent Kit	TaKaRa	Cat# RR064B
SYBR Green	Yeasen Biotechnology	Cat#11201ES03
Mouse IL-1β ELISA kit	Multi Sciences	Cat#EK201B2
Mouse IL-6 ELISA kit	Multi Sciences	Cat#EK206
Mouse TNF-α antibody ELISA kit	Multi Sciences	Cat#EK282
TUNEL assay kit	Keygen Biotech	Cat#KGA704

**Experimental models: Cell lines**		

BV2 cells	Procell Life Science&Technology	CL-0493
POMC neuron	Gift from Prof. Qing-Yan Jiang in South China Agricultural University	N/A

**Experimental models: Organisms/strains**		

C57BL/6 male mice	Shanghai Southern Model Biotechnology Co	N/A
ob/ob male mice	Shanghai Southern Model Biotechnology Co	N/A

**Oligonucleotides**		

Primers, see Table S1	This paper	N/A

**Software and algorithms**		

GraphPad Prism 8	GraphPad Software	N/A
ImageJ	NIH	N/A
Nikon AX	Nikon	N/A
BioRender	BioRender	https://www.biorender.com

### Resource availability

#### Lead contact

Further information and requests for data and resources should be directed to and will be fulfilled by the Lead Contact, Chunxiao Yu (yuchx08@163.com).

#### Materials availability

Materials Availability Statements: This study did not generate new unique reagents.

### Experimental model and study participant details

#### Animals and treatment

All animal experiments were approved by the Animal Ethics Committee of Shandong Provincial Hospital and conducted in accordance with the Animal Care and Use Committee of Shandong Provincial Hospital.

All animals used in the experiments were purchased from Shanghai Southern Model Biotechnology Co. All animals were maintained in a humidity/temperature controlled room (70% relative humidity and 20°C) under an alternating 12 h dark/12 h light cycle and had free access to food and water.

(1) The four-week-old C57BL/6 male mice were randomly divided into two groups: the normal diet-group (n = 9) was fed a normal diet (D12450B, Research Diets, Inc, USA), the high-fat-diet group (n = 16) was fed with high-fat diet (D12492, Research Diets, Inc, USA) for 6 weeks. At the end of the 6 weeks, the mice in high-fat diet group were further subdivided into two subgroups. The first subgroup was fed with high-fat diet and received daily gavage with metformin (∼300 mg/kg body weight/day) (n = 8), the second subgroup maintained the high fat diet and received the same volume of lysed saline gavage (n = 8) for 6 weeks.
(2) Four-week-old C57BL/6 male mice was fed with normal diet as the control group (n = 8). Four-week-old ob/ob male mice were randomly divided into two groups: the drug-administered group (n = 6) was fed with normal diet with daily gavage with metformin (∼300 mg/kg body weight/day), the ob group (n = 8) was fed a normal diet with the same volume of lysed saline gavage for 10 weeks.

At the end of the experiment, all mice were anesthetized, blood was collected, and hypothalamic tissue was quickly removed and frozen in liquid nitrogen until used for assessment of mRNA and protein expression, or fixed in paraformaldehyde for histopathological examination and immunofluorescence staining.

#### Cell culture and co-culture assay

The BV-2 cells line, a mouse microglial cell line, was cultured in Dulbecco’s Modified Eagle Medium (DMEM), supplemented with 10% fetal bovine serum (FBS), streptomycin (100 μg/mL), and penicillin (100 U/mL). The culture supernatant of BV-2 was collected after stimulation. The PA groups were treated with palmitic acid (0.25 mM) treatment and the BSA groups were treated with BSA (175 mg/ml) for 24 h. The PA + Met groups were treated with palmitic acid (0.25 mM) and metformin (1 mM) (Sigma, USA) for 24 h. The culture supernatant of BSA, PA and PA + Met groups was collected after 24 h of stimulation, respectively. Inflammatory cytokine levels in the supernatant were quantified by enzyme-linked immunosorbent assay.

The POMC neurons, a neuronal cell line derived from adult mouse hypothalamic, were seeded into 6-well plates divided into M-BSA, M-PA and M-PM groups and cultured with DMEM containing10% FBS and 1% penicillin–streptomycin. The culture supernatant of BSA, PA and PA + Met BV2 cell groups were added into the medium of M-BSA, M-PA and M-PM POMC neurons groups for 24h, respectively. The POMC neurons were then washed and harvested for subsequent experiments.

### Method details

#### Insulin tolerance test (ITT) and glucose tolerance test (GTT)

For ITT, each mouse was injected intraperitoneally with 1 Unit/kg of euglycemic insulin. For GTT, each mouse in ND, HFD and HFD+Met groups was injected intraperitoneally with 10 μg/g of glucose. The blood glucose levels were measured at 0, 30, 60, 90 and 120 min after insulin or glucose injection using a Roche blood glucose meter respectively.

#### Biochemical analysis

Serum glucose (Glu), total cholesterol (TC), triglycerides (TG), high-density lipoprotein cholesterol (HDL-c), and low-density lipoprotein cholesterol (LDL-c) levels were measured enzymatically using an Olympus AU5400 fully automated biochemical analyzer.

#### Cellular senescence β-galactosidase (SA-β-gal) staining

The brain tissue was fixed by immersion in 4% paraformaldehyde for 24 h and then placed in 10%, 20% and 30% sucrose solutions in sequence for 24 h for dehydration. The brain tissues after sugar immersal were placed in an embedding box, surrounded by the embedding agent OCT, and then frozen to be sliced (approximately 30 μm). The cut frozen sections were first rewarmed and the tissue was washed three times with PBS soaking for 5 min each time. Then, using the Beyoncé SA-β-gal staining kit (Beyotime, C0602), an appropriate volume of β-galactosidase staining fixative was added to fully cover the tissue and fixed at room temperature for 30 min. Wash the tissue 3 times with PBS for 5 min each time. Add the appropriate volume of staining working solution and incubate overnight at 37°C. The next day, capture and process the images with the TISSUEFAXS VIEWER tissue section scanner.

POMC neurons were seeded on a glass slide. The neurons slides were fixed with fixative for 15 min, washed with PBS, then stained with staining working solution for 48 h. Images were acquired using a light microscope.

#### RNA extraction and quantitative real-time fluorescence PCR (qRT-PCR)

Hypothalamic tissue and cell RNA was extracted using Trizol reagent (Takara, Tokyo, Japan). RNA concentration was measured, and then RNA samples were reverse transcribed to obtain cDNA according to the instructions of the reverse transcription kit (One step PrimeScript RT Reagent Kit, TaKaRa, Japan). cDNA was amplified using LC480 (Roche, Mannheim, Germany). cDNA was amplified, and each system consisted of 10 μL SYBR Green (DBI), 1 μL cDNA (5 pmol/μL), 1 μL primer, and 8 μL distilled water. β-actin was used as an endogenous control to normalize the data. The final Cp values were obtained and the relative mRNA expression levels were calculated using the 2-ΔΔCt method. Primer sequences were listed in Table S1.

#### Protein extraction and immunoblot analysis

Hypothalamic tissues or cells were homogenized in protein lysis buffer (RIPA: PMSF = 99:1) (sample: protein lysis solution = 1:15) and then subjected to ultrasonic lysis, followed by centrifugation for 15 min. Protein content was determined using a BCA kit (Shanghai Shenergy Gaming). After denaturation, protein samples were subjected to SDS-PAGE and electrophoretically transferred to PVDF membranes. The membranes were closed in 5% skimmed milk (prepared with TBST) for 1 h and then incubated overnight at 4°C with specific antibodies, including those related to inflammation, i.e., TNFα, IL-6, NLRP3, CASPASE1 and STING; ER-related signaling pathway proteins, i.e., p-PERK, t-PERK, BIP, *p*-eIF2α, t eIF2α, ATF4, p-IRE1α, t-IRE1α, and sXBP1; and β-actin as an internal reference. After washing three times in TBST, the membranes were incubated with secondary antibodies for 1 h. Protein bands were visualized using an Amersham Imager 680 (AI 680, GE, USA) ultrasensitive chemiluminescence imager. The band density was quantified using Alphaview-FluorChen Q SA software.

#### Immunofluorescence staining

For the immunofluorescence assay, the frozen sections and cell slides were closed with 5% donkey serum protein for 30 min, followed by the addition of specific primary antibodies incubated overnight at room temperature, namely BIP (1:1000), IBA1 (1:400), STING (1:400), P16 (1:200), γ-H2AX (1:300), Ki67 (1:500) and NeuN (1:200). The next day, the tissues were then incubated with the donkey anti-rabbit IgG (H + L) Alexa Fluor 555, donkey anti-mouse IgG (H + L) Alexa Fluor 488, donkey anti-mouse IgG (H + L) Alexa Fluor 555, or donkey anti-goat IgG (H + L), Alexa Fluor 488 (Thermo Fisher Scientific, USA) at a 1: 1000 dilution at room temperature for 1 h. After washing with PBS, cell nucleus was stained with DAPI. Nikon AX (Japan) fluorescence confocal microscope was used to capture and process the images. Images were analyzed using ImageJ software. For descriptive analyses, each sample was examined at least 3 times.

#### IL-6, IL-1β and TNF-α Measurements

Assay kits for IL-6, IL-1β and TNF-α were provided by Multi Sciences (China). The levels of IL-6, IL-1β and TNF-α were measured using the kits according to the manufacturer’s instructions.

#### Terminal deoxynucleotidyl transferase dUTP nick end labeling (TUNEL) assay

The frozen sections and cells on cover slips were stained using a TUNEL assay kit (KGA704-1; Keygen Biotech Co., Ltd., Nanjing, China), according to the manufacturer’s protocol. The cell nuclei were stained with DAPI and the sections were observed using the Nikon AX (Japan) fluorescence confocal microscope. Each sample was assessed at least 3 times.

### Quantification and statistical analysis

All data were analyzed using GraphPad Prism (version 8, GraphPad Software Inc, CA). An unpaired t-test was used to compare means between two groups, and a one-way ANOVA was used to compare means among multiple groups. The data are shown as the mean ± SEM. A value of p < 0.05 or 0.01 was considered statistically significant. *In vivo* experiments, ∗p < 0.05, ∗∗p < 0.01 means metformin or HFD+Met group compared to control or ND group; #p < 0.05, ##p < 0.01 means metformin or HFD+Met group compared to vehicle or HFD group. *In vitro* experiments,∗p < 0.05, ∗∗p < 0.01 means PA + Met or M-PM group compared to BSA or M-BSA group; #p < 0.05, ##p < 0.01 means PA + Met or M-PM group compared to PA or M-PA group.

## Data Availability

• All data reported in this paper will be shared by the lead contact upon request. • This paper does not report original code. • Any additional information required to reanalyze the data reported in this paper is available from the lead contact upon request.
