# Supplementary material for: Metformin inhibits inflammatory response and endoplasmic reticulum stress to improve hypothalamic aging in obese mice
Source: iScience. 2023 Sep 27;26(10):108082. doi: 10.1016/j.isci.2023.108082 (PMC10582490; doi:10.1016/j.isci.2023.108082)
Supplement: Document S1. Figures S1 and S2 and Table S1 [file mmc1.pdf]

iScience, Volume *26*

Supplemental information

Metformin inhibits inflammatory response and endoplasmic reticulum stress to improve hypothalamic aging in obese mice

Leilei Yang, Peng Lu, Xiangyu Qi, Qian Yang, Luna Liu, Tao Dou, Qingbo Guan, and Chunxiao Yu

Supplemental Information

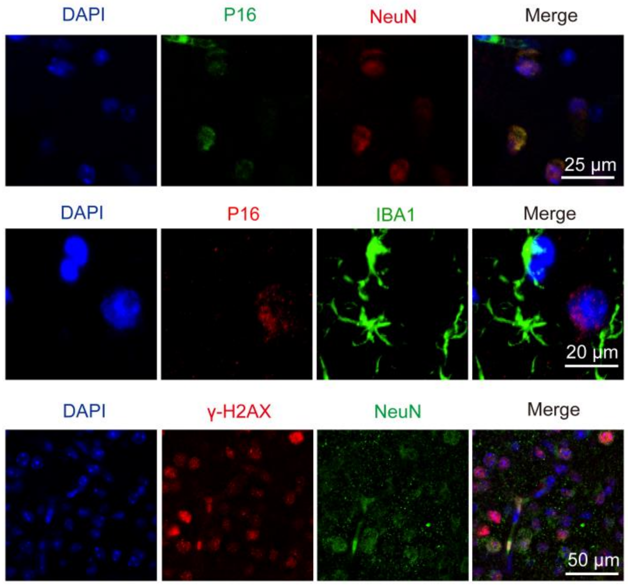

Figure S1. Expression analysis of aging marker in neuron and microglia, related to Figure 2.

Co-immunofluorescence staining of P16 with NeuN and IBA1, and co-immunofluorescence staining of γ-H2AX with NeuN in obese mice. Bar means 25, 20 or 50 μm.

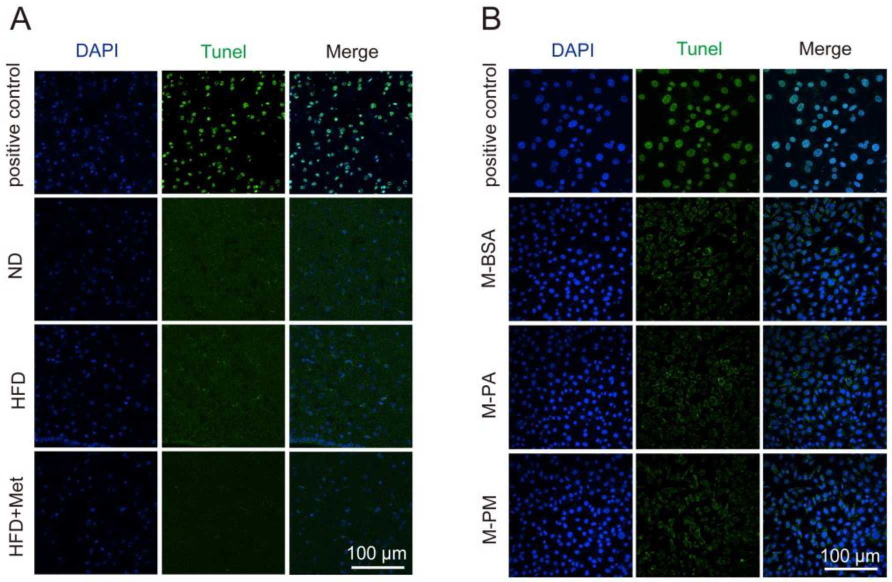

Figure S2. Apoptosis analysis of hypothalamus and neurons, related to Figure 2.

A.) Tunel staining of hypothalamus in high-fat diet induced obese mice after metformin treatment. Bar means 100 μm.

B.) Tunel staining of POMC neurons treated with BV2 cell culture supernatant treated with palmitic acid and metformin. Bar means 100 μm.

Table S1. The lists of primers used in qRT-PCR, related to STAR Methods.

Gene name	Gene ID	Forword	Reverse
p16	12578	CTTGGTGAAGTTCGTGCGATCC	GCACCGTAGTTGAGCAGAAGAG
h2ax	15270	CGGTGGGCTTGAAGGTTAGT	ACTGGTATGAGGCCAGCAAC
Bip	14828	TCTCAGCATCAAGCAAGG	ATCCACCAACCAGAACAAT
Perk	13666	ACTCCAATGCCAGCCTATGC	CAAGCCTAAAGAGAAGATGTCCACT
eif2α	13665	AGCCACATCCAGGAAGTGACAA	ACAGGAGTAGGAGCCGCATCA
Atf4	11911	GCCAAGCACTTGAAACCTCA	CCATTTTCTCCAACATCCAATC
Chop	13198	CTGGAAGCCTGGTATGAGGAT	CAGGGTCAAGAGTAGTGAAGGT
Ire1α	78943	AATCAGACGAGCACCCAAATG	CTGCTCCACATACTCTTGTAGGGT
xbp1	22433	ACGCTTGGGAATGGACACG	GGGAAGATGTTCTGGGGAGG
Atf6	226641	TCGCCTTTTAGTCCGGTTCTT	GGCTCCATAGGTCTGACTCC
Tnfα	21926	CCCTCACACTCAGATCATCTTCT	GCTACGACGTGGGCTACAG
Il-1β	16176	TGGACCTTCCAGGATGAGGACA	GTTCATCTCGGAGCCTGTAGTG
Il-6	16193	TAGTCCTTCCTACCCCAATTTCC	TTGGTCCTTAGCCACTCCTTC
Il-18	16173	GACTCTTGCGTCAACTTCAAGG	CAGGCTGTCTTTTGTCAACGA
Sting	72512	GGTCACCGCTCCAAATATGTAG	CAGTAGTCCAAGTTCGTGCGA
Nlrp3	216799	ATTACCCGCCCGAGAAAGG	TCGCAGCAAAGATCCACACAG
Caspase1	12362	TTGAAAGACAAGCCCAAGGTG	CTGGTGTTGAAGAGCAGAAAGC
Asc	66824	CTGGAGTCGTATGGCTTGGAG	CAAAGTGTCCTGTTCTGGCTGTA
β-actin	11461	GGCTGTATTCCCCTCCATCG	CCAGTTGGTAACAATGCCATGT
